# Enhanced Tolerance to Antifungals as a General Feature of Rho^−^ Mutants in Yeast Species: Implications to Positive Selection of Respiratory Deficiency

**DOI:** 10.3390/microorganisms13010099

**Published:** 2025-01-07

**Authors:** Zachary Johnson, Farhan Nadim, Mikhajlo K. Zubko

**Affiliations:** Centre for Bioscience, Manchester Metropolitan University, John Dalton Building, Chester Street, Manchester M1 5GD, UK; zacharyjohnsn@gmail.com (Z.J.); farhannadim72@outlook.com (F.N.)

**Keywords:** antifungals, azoles, *Candida*, *Saccharomyces*, rho^−^ mutants, petite, positive selection, tolerance, sensitivity, disc diffusion

## Abstract

Although the mitochondrial genome is an attribute of all eukaryotes, some yeast species (called petite-positive) can replicate without mitochondrial DNA (mtDNA). Strains without mtDNA (known as rho^−^ mutants or petite mutants) are respiration-deficient and require fermentable carbon sources (such as glucose) for their metabolism. However, they are compromised in many aspects of fitness and competitiveness. Nevertheless, a few research groups have reported that some petite mutants of *Candida glabrata* and *Saccharomyces cerevisiae* manifested higher levels of tolerance to the antifungal fluconazole than their wild-type (WT) counterparts. In this study, we show that elevated tolerance to two or three out of four tested antifungals is a generic feature of at least five petite-positive species of yeasts including *C. glabrata* (higher tolerance of petites to clotrimazole and miconazole), *S. bayanus* (tolerance to clotrimazole, fluconazole, and miconazole), *S. cerevisiae* (tolerance to clotrimazole and fluconazole), *S. paradoxus* (tolerance to clotrimazole, fluconazole, and miconazole), and *S. pastorianus* (tolerance to clotrimazole and fluconazole). Comparing the levels of tolerance to the antifungals in WT and petite mutants was based on measuring the diameters of the zones of inhibition (ZOIs) using disc diffusion assays. The mode of inhibition in the majority of WT strains by all antifungals was fungicidal; most of the rho^−^ mutants manifested fungistatic inhibition. We observed partial (not complete) inhibition in WT, with four different types of ZOI patterns that were species- and antifungal-specific. The partial inhibition was characterised by the presence of antifungal-tolerant colonies within ZOI areas. The inability of these colonies selected from ZOIs to grow on glycerol, as a single source of carbon, proved that they were rho^−^ mutants spontaneously generated in the WT populations. The results on the elevated tolerance of petite strains to antifungals are discussed in terms of the prospective positive selection of respiratory-deficient mutants and the various implications of such selection.

## 1. Introduction

Possessing the mitochondrial genome (in addition to nuclear genome) is a generic feature of all eukaryotes including higher organisms and eukaryotic microorganisms [1,2,3,4,5]. Mitochondrial DNA (mtDNA) encodes an important for life set of OXPHOS enzymes essential for respiration. Mutations in mitochondrial genes for the OXPHOS machinery affect the functionality of respiratory enzymes and therefore compromise or entirely abolish the respiratory capacity of cells, making them respiratory-deficient. Mutations in genes encoding mitochondrial tRNA and rRNA weaken translation in mitochondrial ribosomes and overall energy production in mitochondria, which are reasons why many mutations in mtDNA cause diseases in humans [6,7,8]. A substantial volume of knowledge about the biology of mitochondria comes from studying the budding yeast *Saccharomyces cerevisiae*, which remains a favourite model system [4]. Some yeast species with fermentation metabolism (in addition to respiration) can thrive without mtDNA if a fermentable carbon source (such as glucose) is available [5,9]. Yeast strains deficient in mtDNA are known as petite mutants (because of small size of their colonies) or rho^−^ mutants. Rho^−^ mutants with completely deleted mtDNA are called rho0 mutants. Yeast species able to produce viable rho^−^ mutants are named petite-positive and include some species from the genera *Brettanomyces*, *Candida*, *Kloeckera*, *Saccharomyces*, and *Torulopsis* [9,10].

The existence of petite-positive yeast cells partially or completely lacking mitochondrial DNA provides a unique model for studying the roles of mtDNA in different biological processes [5] as well as for understanding nucleo–mitochondrial interactions [11]. The petite phenotype of rho^−^ mutants itself implies that their general metabolic fitness and adaptation to environmental perturbations are reduced [12]. However, not much is known about the fitness and adaptability of rho^−^ mutants under various stress conditions that could mimic aetiological factors in pathogenesis, environmental competitiveness, and potentially evolutionary forces. Nevertheless, such knowledge would be beneficial for understanding the pathology of fungal diseases, environmental and population genetics, and evolutionary biology. Despite the volume of research regarding these aspects being limited overall, some studies of this sort have been initiated. Previously, it was shown that rho^−^ mutants are compromised in their tolerance to many factors including hydrogen peroxide [13,14], sodium chloride [15], 4-nitroquinoline 1-oxide [14], elevated temperatures [16], and the anticancer drug 3-bromopyruvate [17]. These observations suggest an involvement of the mitochondrial genome of yeast species (and potentially other eukaryotes) into evolutionary adaptation to environmental stress [15,16,18,19]. Broadening this aspect led us to enquire as to what other factors could potentially be covered by the evolution of mtDNA. In particular, an attractive point of interest would be to look at the stress factors to which rho^−^ mutants demonstrate more resilience than their wild-type counterparts. In the last three decades, there have been a few sporadic medically-orientated publications showing the resistance of some yeast strains to azole derivatives, with the observed links to their respiratory deficiency or petite phenotypes in *C. glabrata* [20,21,22,23] as well as in *S. cerevisiae* [24]. These and other similar findings stimulated us to undertake a systematic comparative study to establish under uniform experimental settings whether the enhanced tolerance of rho^−^ mutants to antifungal drugs is a general feature of various petite-positive yeast species and to discuss the implications of this enquiry at a broader scale than it was previously carried out.

## 2. Materials and Methods

### 2.1. Yeast Strains and Media Used

Wild type (WT) and rho^−^ mutants of five petite-positive species were used (Table 1). Molecular analyses for their rho^−^ status have been described in our previous work [16]. Taken from frozen glycerol stocks at −80 °C, the WT strains were initially grown on YEPG solid medium containing 3% glycerol as a single carbon source (to eliminate possible respiratory-deficient mutants). All rho^−^ mutants were initially revived and maintained further on solid YEPD medium. Sensitivity testing for the WT and rho^−^ strains was performed using YEPD plates. Both media are described in [25]. The incubation temperature for all cultures was usually 30 °C. Wild-type strains of *Candida glabrata, Saccharomyces bayanus, S. cerevisiae, S. paradoxus*, and *S. pastorianus* were grown on agar containing YEPD and YEPG.

### 2.2. Sensitivity Testing

To compare the tolerance levels of the WT and rho^−^ mutants to antifungal drugs, freshly grown cells were resuspended in sterile water to the density of the 0.5× McFarland standard, and swabbed onto YEPD plates. Commercial discs containing amphotericin B (20 µg), clotrimazole (10 µg), miconazole (10 µg) [Mast Group Ltd., Bootle, UK], and fluconazole (25 µg) [Fisher Scientific, Loughborough, UK] were applied onto the surface of the agar (with sterile forceps) and pressed slightly for good contact with the agar. Plates were incubated for two days at 30 °C. Each disc diffusion assay was conducted in at least three replicas. Following the incubation, diameters of the zones of inhibition (ZOIs) were measured, and plates were photographed near a ruler.

### 2.3. Replica Plating

Replica plating of colonies grown within the ZOIs (around discs with antifungals) was conducted using sterile velvet tissues fixed on the top of a rubber block (to replicate areas with diameter 9 cm), or on a capped plastic universal (to replicate smaller areas, up to 3 cm in diameter). In the latter case, the universal itself was used as a handle, and the cap served as a platform for fixing velvet tissues with rubber bands. The colonies were replicated onto plates containing solid YEPD and YEPG media. More details of this technique can be found in a classic paper by J. Lederberg and E.M. Lederberg [26].

### 2.4. Spot Tests

In addition to sensitivity testing via disc diffusion, spot test assays were employed to determine how different concentrations of fluconazole in media affected the growth of WT and rho^−^ mutants of different species. A stock solution of fluconazole (Merck Life Science UK Limited, Gillingham, UK) was prepared at the concentration of 10 mg/mL in DMSO and added to autoclaved, aliquoted, and cooled to 60 °C YEPD and YEPG media to final concentrations of 0, 16, 32, and 64 µg/m. The aliquots were poured onto plates to solidify. Spot tests were performed as previously described [16].

### 2.5. Mode of Inhibition Assays

To test the mode of inhibition (MoI), a simple technique based on the survival levels of the inhibited cells was employed [27]. In brief, if cells rubbed from ZOIs produced substantial growth on the antifungal-free YEPD agar, it was interpreted as a fungistatic effect. No survival (or poor survival) indicated fungicidal MoI. To minimise misinterpretation due to the possible presence of invisible contaminants or resistant mutants within the ZOIs, at least three different locations from each ZOI were tested independently. Replica plating of ZOIs to YEPD and YEPG plates was an alternative approach to assess the MoI. As an advantage of this method, the replication from whole plates was faster and more informative because of the coverage of the entire ZOI.

### 2.6. Data Analysis

All tests were performed in at least three (or more) replicas. Calculation of the mean ± SEM was complemented by using the Mann–Whitney U test, Kruskal–Wallis test, and Dunn’s post hoc test with Bonferroni correction.

## 3. Results

### 3.1. Respiratory Deficient Rho^−^ Mutants of Petite-Positive Yeast Species Are More Tolerant to Some Antifungals than Their WT Counterparts

**Figure 1:**
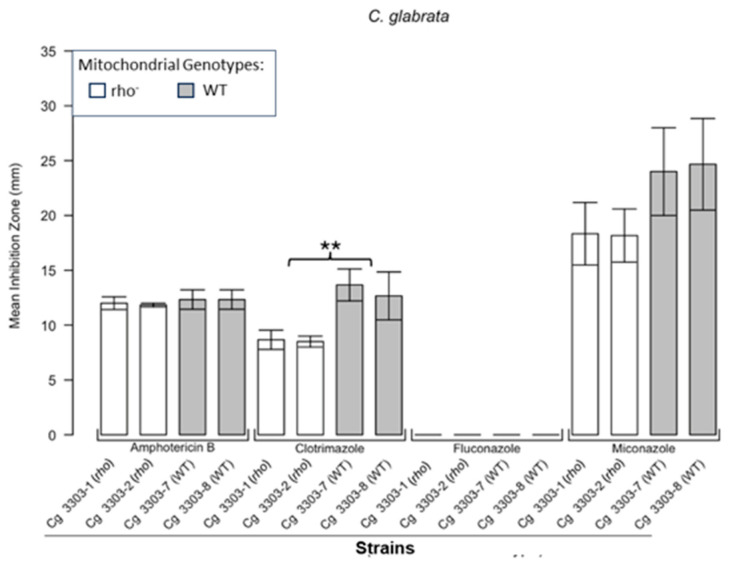
**Inhibition of *C. glabrata* by four antifungals in the disc diffusion assays.** Bar plots present the average ZOI diameters for two WT strains (Cg 3303-7, Cg 3303-8) and two rho^−^ strains (Cg 3303-1, Cg 3303-2) for the same antifungals. Error bars represent the standard error of the mean (SEM). Significant differences are indicated for the Mann–Whitney U results (** *p* < 0.01). Data are presented as the mean ± SEM from three independent assays (n = 3).

**Figure 2:**
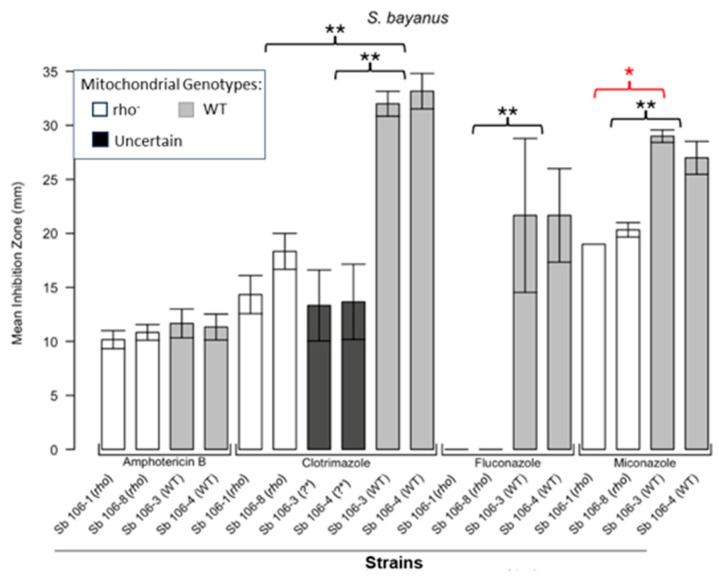
**Inhibition of *S. bayanus* by the four antifungals in the disc diffusion assays.** Bars represent the ZOIs in the WT strains (Sb 106-3, Sb 106-4) and two rho^−^ strains (Sb 106-1, Sb 106-8) as well as the anomalous ZOIs for clotrimazole: Sb 106-3 (?*) and Sb 106-4 (?*). Significant differences are indicated for the Mann–Whitney U results in black and Kruskal–Wallis results (Dunn’s post hoc test and Bonferroni correction) in red (* *p* < 0.05, ** *p* < 0.01). Data are presented as the mean ± SEM from three independent assays (n = 3).

**Figure 3:**
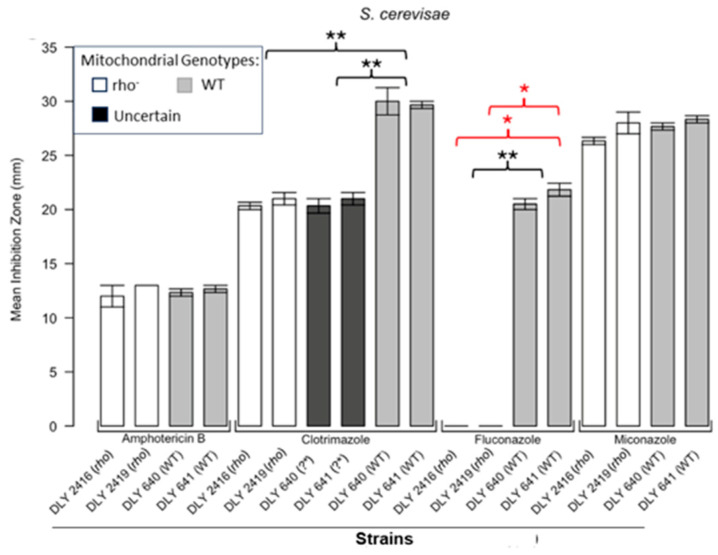
**Inhibition of *S. cerevisiae* by the four antifungals in the disc diffusion assays.** Bars represent the ZOIs in the WT strains (DLY 640, DLY 641) and two rho^−^ strains (DLY 2416, DLY 2419) as well as the anomalous ZOIs for clotrimazole: DLY 640 (?*) and DLY 641 (?*). Error bars represent the SEM. Significant differences are indicated for the Mann–Whitney U results in black and Kruskal–Wallis results (Dunn’s post hoc test and Bonferroni correction) in red (* *p* < 0.05, ** *p* < 0.01). Data are presented as the mean ± SEM (n = 3).

**Figure 4:**
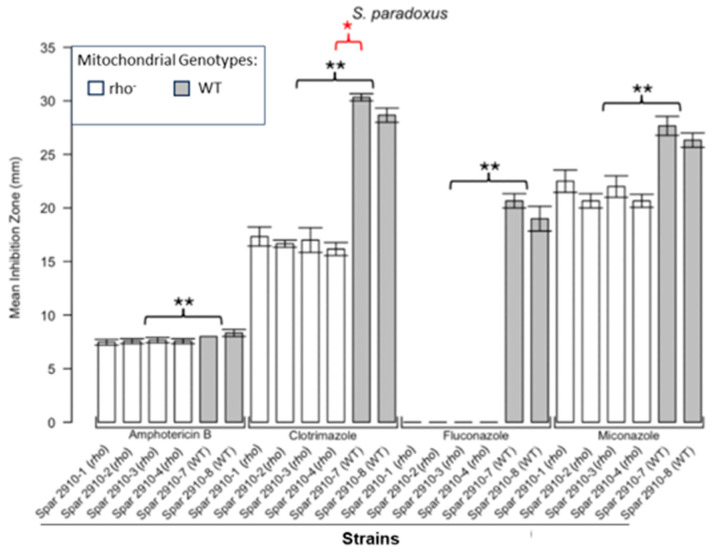
**Inhibition of *S. paradoxus* by the four antifungals in the disc diffusion assays.** Bars represent the ZOIs in the WT strains (Spar 2910-7, Spar 2910-8) and four rho^−^ strains (Spar 2910-1, Spar 2910-2, Spar 2910-3, Spar 2910-4). Error bars represent the SEM. Significant differences are indicated by the Mann–Whitney U results in black and Kruskal–Wallis results (Dunn’s post hoc test and Bonferroni correction) in red (* *p* < 0.05, ** *p* < 0.01). Data are presented as the mean ± SEM (n = 3).

**Figure 5:**
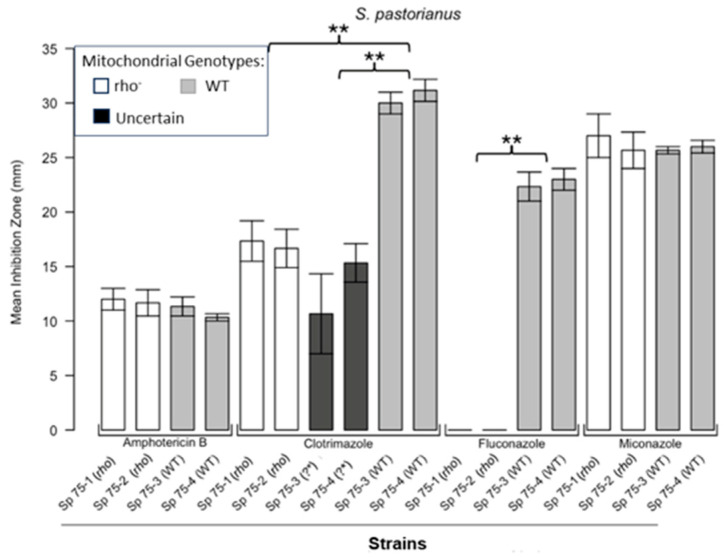
**Inhibition of *S. pastorianus* by the four antifungals in the disc diffusion assays.** Bars represent the ZOIs in two WT strains (Sp 75-3, Sp 75-4) and two rho^−^ strains (Sp 75-1, Sp 75-2) as well as the anomalous ZOIs for clotrimazole: Sp 75-3 (?*) and Sp 75-4 (?*). Error bars represent the SEM. Significant differences are indicated for the Mann–Whitney U results (** *p* < 0.01). Data are presented as the mean ± SEM (n = 3).

In a preliminary experiment (Supplementary Figure S1), we observed pronounced differences in the responses of the WT and rho^−^ mutants of *Saccharomyces cerevisiae* to clotrimazole and fluconazole. Two rho^−^ mutants of independent origin were clearly more tolerant to these antifungals than the WT strains. This observation initiated our enquiry as to whether higher tolerance to the antifungals is a common feature of rho^−^ mutants of other petite-positive species, or not. A systematic study to answer this question began by comparing responses of wild type (WT) and rho^−^ mutants of *Candida glabrata*, *Saccharomyces bayanus*, *S. cerevisiae*, *S. paradoxus*, and *S. pastorianus* to four antifungals, namely amphotericin B, clotrimazole, fluconazole, and miconazole, all impregnated in discs. Each species was presented by at least four strains—two isogenic genotypes of independent origin for both the WT and rho^−^ mutants. All used strains were previously described [16]. The initial growth of all WT strains on YEPG medium was undertaken to purify their populations from spontaneous respiratory-deficient mutants. Images in Supplementary Figure S2 represent examples of primary data from these experiments. Averaged diameters of ZOIs from at least three replicas for each strain are presented in Figure 1, Figure 2, Figure 3, Figure 4 and Figure 5.

Figure 1 shows the results for the WT and rho^−^ mutants of *C. glabrata*. These were not inhibited by fluconazole, but were inhibited to the same extent with amphotericin B. The rho^−^ mutants demonstrated significantly more tolerance to clotrimazole than their WT counterparts. The rho^−^ strains also manifested a consistent trend of higher tolerance to miconazole, although this tendency was not statistically significant. The overall inhibition patterns for the WT and rho^−^ mutants of *S. bayanus* (Figure 2), *S. cerevisiae* (Figure 3), *S. paradoxus* (Figure 4), and *S. pastorianus* (Figure 5) were the same or very similar. Petite mutants of the four species manifested no inhibition by fluconazole, while the correspondent WT strains were strongly inhibited, with ZOIs between 20 and 23 mm in diameter. Inhibition with amphotericin B did not differ for the rho^−^ mutants and WT of *S. bayanus* (Figure 2), *S. cerevisiae* (Figure 3), and *S. pastorianus* (Figure 5), with the ZOI diameters ranging between 10 mm and 13 mm. ZOIs caused by amphotericin B in *S. paradoxus* (Figure 4) were sized between 7.5 mm and 8.5 mm, showing a statistically slightly higher tolerance of rho^−^ mutants compared with the WT.

All four species demonstrated a significantly higher tolerance of their rho^−^ mutants to clotrimazole, with 30–60% smaller ZOI diameters than in the WT strains. Petite mutants of two species, *S. bayanus* (Figure 2) and *S. paradoxus* (Figure 4), showed a significantly higher tolerance to miconazole than was seen for their WT counterparts.

### 3.2. Variations of ZOI Patterns Are Strain- and Drug-Dependent

**Figure 6:**
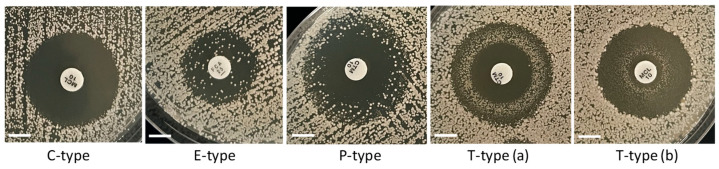
**Types of ZOI patterns observed for partial inhibition in the disc diffusion assays.** Scale bar = 6 mm.

We observed four types of ZOI patterns depicted in Figure 6 and Supplementary Figure S2. For simplicity, we abbreviated these types using capital letters associated with the main characteristics of the ZOIs in each case. The first type denoted as C-type (“clear”) is represented by ZOIs with clear areas and relatively sharp edges. All ZOIs found in the rho^−^ mutants were of this type. Three other types of ZOIs resulted from not complete (partial) inhibition and contained different numbers of colonies inside the main ZOIs, with various distributions of those colonies within ZOI areas. In the case of E-type (“even”), colonies were evenly distributed within the main ZOI. Such ZOI patterns were typically produced by fluconazole inhibiting the WT strains of all *Saccharomyces* species. P-type (“peripheral”) was presented by partial inhibition with the predominant abundance of extra colonies at the periphery of ZOIs. The T-type (“torus”) of ZOIs manifested the accumulation of extra colonies in a torus-like ring between the disc and the edge of the ZOI. We observed the rings situated either at the periphery of the ZOI [T-type (a)] or closer to the disc [T-type (b)].

Since the partial inhibition was observed only for the WT strains, we reasoned that it could reflect the presence of spontaneously generated rho^−^ mutants within populations of WT. Therefore, in the next experiments, we aimed to relate the incidence of colonies in zones of partial inhibition to the status of respiratory deficiency.

### 3.3. Incomplete Inhibition Outcomes Predominantly from Spontaneous Generation of Respiratory-Deficient Cells with Enhanced Tolerance to Antifungals

For verification that the incomplete inhibition patters resulted from the presence of some proportion of respiratory-deficient cells in the WT populations, ZOIs obtained for all four antifungals against *S. bayanus, S. cerevisiae*, and *S. pastorianus* were replicated to YEPD and YEPG (Supplementary Figure S3) using a replicator with sterile velvet tissues [26]. These assays helped to observe the overall landscape of growth/no growth on YEPD and YEPG throughout all areas of the ZOIs, thus to conclude about the status of the respiratory proficiency/deficiency of bulk colonies formed within the ZOIs. In addition, the lack of growth on both YEPD and YEPG media implied fungicidal mode of inhibition. This was the case for the amphotericin B inhibition of all three species as well as the inhibition of WT *S. cerevisiae* by miconazole and clotrimazole. Most colonies replicated from ZOIs produced by clotrimazole, fluconazole, and miconazole in *S. bayanus* did not grow further on YEPG, indicating their respiratory deficiency. All colonies of *S. cerevisiae* from the fluconazole ZOIs possessed the same status of deficiency. Colonies of *S. pastorianus* on the ZOIs induced by clotrimazole and miconazole were also respiratory-deficient. Interestingly, numerous colonies of *S. pastorianus* that appeared on fluconazole ZOIs grew well on YEPG, suggesting their respiratory proficiency. This could be explained by the enhanced survival of WT cells after inhibition by fluconazole. Overall, these results show that partial inhibition accompanied by various ZOI patterns could be explained by the presence of respiratory-deficient mutants that are more tolerant to antifungals in WT populations.

### 3.4. Modelling Experiments Towards Positive Selection of Respiratory-Deficient Mutants

**Figure 7:**
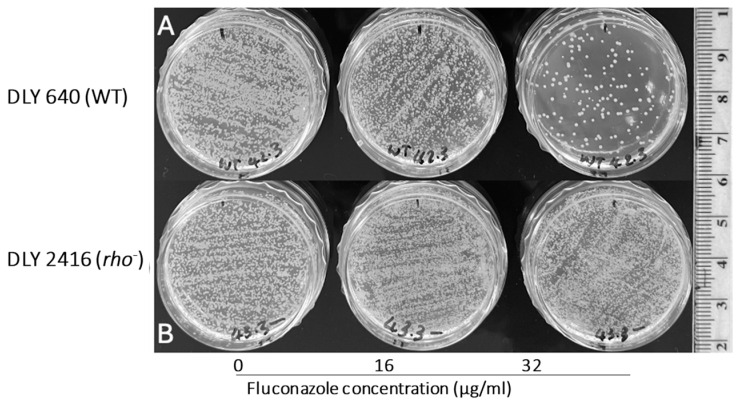
**Comparison of growth for the WT and rho^−^ mutants of *S. cerevisiae* under fluconazole supplementation.** (**A**, top row) Growth of the WT DLY 640 strain. (**B**, bottom row) Rho^−^ strain DLY 2416 on YEPD media supplemented with 0, 16, or 32 µg/mL of fluconazole. Each condition was tested in three independent biological replicates, with the results shown as being representative of these runs.

**Figure 8:**
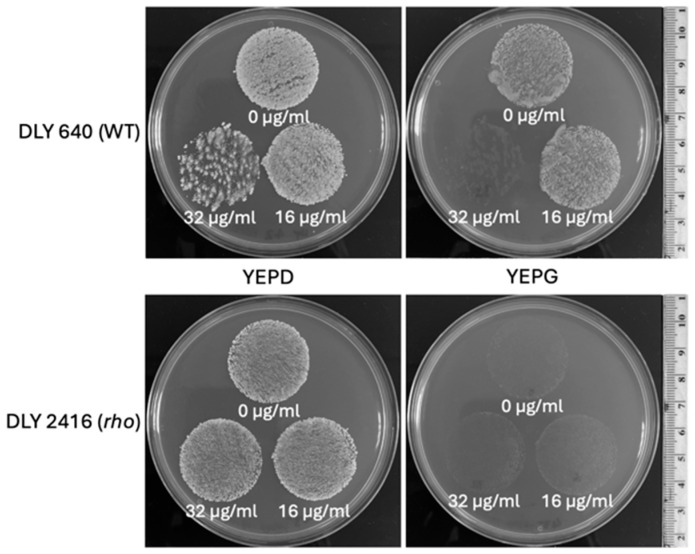
**Replica plating of the WT and rho^−^ populations of *S. cerevisiae* from fluconazole-supplemented media.** The top row shows the WT strain DLY 640. The bottom row shows the rho^−^ strain DLY 2416, corresponding to the populations shown in Figure 7. Populations were replica plated onto fresh YEPD (**left**) and YEPG (**right**) plates. The corresponding fluconazole concentrations were labelled below the respective population replica. Each of the independent replicates presented in Figure 7 were replica plated. The images shown here are representative of these runs.

The enhanced tolerance of rho^−^ mutants to some antifungals implies a possibility for positive selection of respiratory-deficient cells in the presence of the tested inhibitors. To examine this directly, we plated suspensions of a WT and a rho^−^ mutant of *S. cerevisiae* cells (at the density of 0.5× McFarland) on YEPD medium in the presence of fluconazole at a few concentrations to find the inhibiting concentrations for the WT. Figure 7, depicting a part of this experiment, shows that the MIC of fluconazole for the WT was between 16 µg/mL and 32 µg/mL. At the concentration of 16 µg/mL, the density of grown colonies in the WT was apparently reduced compared with the bulk on YEPD without fluconazole. The density of the rho^−^ mutant colonies remained the same for both fluconazole concentrations and the control without fluconazole. A low proportion of single colonies grown within the WT population at 32 µg/mL of fluconazole were presumably respiratory-deficient (most likely rho^−^ mutants). To verify this, the plates were replicated onto YEPD and YEPG media (Figure 8). All colonies initially grown from the WT population at 32 µg/mL of fluconazole were not able to produce any growth after replication on the YEPG medium, thus confirming their status of respiratory deficiency. This assay also allowed us to draw an additional conclusion that the inhibition of WT cells on the medium containing 32 µg/mL of fluconazole was fungicidal. Replication of WT cells from fluconazole at the concentration of 16 µg/mL resulted in confluent growth on YEPG, suggesting that the growing cells originated from respiratory-proficient WT. Overall, it can be concluded that fluconazole at the concentration of 32 µg/mL (or similar) provides a convincing selective background for 100% recovery of respiratory-deficient clones from the WT population.

We observed that some proportions of the colonies grown in the presence of 32 µg/mL of fluconazole were of a smaller size than the majority in the bulk (Figure 7, plate at the top right). Thinking about the possible relation of this observation to the rho status of the colonies, we re-suspended twenty colonies (10 small and 10 large) in small volumes of water (50 µL) and patched the suspensions in arrays onto YEPD and YEPG agar plates to assess their growth after 2 days of incubation at 30 °C. All twenty isolates grew well on YEPD medium but did not grow on YEPG (Supplementary Figure S4), suggesting that all tested colonies were respiratory-deficient. Therefore, the reason for the different colony size remains unclear.

### 3.5. Modes of Inhibition of Yeast Cells by Antifungals

Modes of inhibition for each antifungal were assessed directly from the ZOIs by streaking out or by replicating inhibited cells from the ZOIs to media free of antifungals. In both approaches, the regrowth of cells on antifungal-free media was interpreted as fungistatic MoI, while no growth was referred to as fungicidal MoI. Examples of streaking out from the ZOIs for *C. glabrata* are shown in Supplementary Figure S5. In these assays, only the amphotericin B results were consistent with fungicidal inhibition. Very soon after applying the streaking approach, we realised that incomplete inhibition with various patterns of ZOIs observed in many cases (Figure 6 and Supplementary Figure S2) limited the interpretation of results on MoI obtained by streaking out from the ZOIs. The main reason for this was the presence of resistant colonies in the ZOIs, which should inevitably regrow in the MoI assay. To overcome this limitation, we attempted another approach to assess MoI based on the replica plating of ZOIs to YEPD and YEPG media. This alternative technique should allow for an analysis of the inhibitory effects in different areas of the ZOIs at the same time, and it is less dependent on the presence of colonies in the ZOIs. The results of replica plating of the ZOIs from testing the WT cells are presented in Supplementary Figures S6–S10 and are summarised in Supplementary Table S1. The presence of rho^−^ mutants in the WT populations allowed us to make extrapolations regarding the inhibition modes in respiratory-deficient cells. The MoI of amphotericin B was fungicidal for the WT of *C. glabrata*, *S. bayanus*, *S. cerevisiae*, and *S. pastorianus*. The results regarding this antifungal were not conclusive for rho^−^ mutants of all species but *S. pastorianus* whose respiratory-deficient cells from the pool of the WT manifested fungicidal MoI. The WT of all four species responded in the same mode (fungicidal) to clotrimazole. Petites of these species showed static inhibition by clotrimazole. Fluconazole inhibited the WT of *S. cerevisiae* in cidal mode. The WT cells of all other species were inhibited statically. The response of *C. glabrata* rho^−^ cells was also in static mode. The data for the rho^−^ mutants of all other species were not conclusive. The MoI by miconazole for the WT of all five species was fungicidal; for the rho^−^ mutants of *C. glabrata, S. cerevisiae,* and *S. pastorianus*, it was static; for the rho^−^ cells of *S. paradoxus*, the MoI was cidal; and for *S. bayanus*, the data were not conclusive. In summary, generic responses of the WT cells of all five species to the four tested antifungals were fungicidal, while the overall responses of the rho^−^ cells were fungistatic. This is consistent with and complementary to the data obtained via the disc diffusion assays, showing generally enhanced tolerance of rho^−^ mutants to the tested antifungals compared with the WT.

## 4. Discussion

In this study, we showed that enhanced tolerance to a few antifungals is a general feature of rho^−^ mutants in yeast petite-positive species, in particular *C. glabrata, S. bayanus, S. cerevisiae, S. paradoxus*, and *S. pastorianus.* The choice of these species was based on their availability in our collections and on the characterisation of these strains in our previous work [16]. Disc diffusion assays based on the analysis of ZOIs were sufficient to demonstrate that all rho^−^ mutants of *Saccharomyces* were entirely resistant to fluconazole and manifested a higher tolerance to clotrimazole than their WT counterparts. Despite there being no obvious differences in the responses of most of the rho^−^ mutants and WT to amphotericin B, *S. paradoxus* mutants were statistically slightly more resilient to this antifungal than the WT. *S. bayanus* and *S. paradoxus* rho^−^ mutants showed enhanced tolerance to miconazole. In general, rho^−^ mutants of *S. paradoxus* (Figure 4) showed statistically higher tolerance to all four antifungals than their WT counterparts. *C. glabrata* rho^−^ mutants were more tolerant to clotrimazole and miconazole than the WT. Interestingly, their WT and rho^−^ strains were equally resistant to fluconazole. It was previously shown that petites of *C. glabrata* were fluconazole resistant [21,23]. We reasoned that differences in susceptibility between the WT and rho^−^ mutants of *C. glabrata* should be detectable at higher concentrations of fluconazole. Spot tests following serial dilutions of yeast populations demonstrated the possibility of regulating the growth responses and therefore the stringency of selection using different concentrations of the drug (Supplementary Figure S11). In this experiment, the WT and rho^−^ strains of *C. glabrata* manifested the same levels of growth in the presence of 16 µg/mL and 32 µg/mL of fluconazole, but at the concentration of 64 µg/mL, the rho^−^ cells grew better than the WT. These results could be linked to observations from a study showing elevated survival rates of *C. glabrata* petites engulfed by macrophages and not killed by antifungal drugs [28]. Since *C. glabrata* is becoming a high-priority pathogen causing candidemia in recent years [29], these data should stimulate the development of new antifungal drugs that are efficient at treating rho^−^ mutants that are always present in *C. glabrata* infections.

Azoles target the synthesis of ergosterol in fungal membranes. They inhibit the cytochrome P450 lanosterol 14-α-demethylase, an enzyme for the biosynthesis of ergosterol [30]. It has been shown that in the presence of fluconazole, mitochondria mediate the conversion of non-toxic 14α-methyl fecosterol into toxic 14α-methyl sterols. Petite mutants have dysfunctional mitochondria, thus preventing the accumulation of toxic sterols and promoting tolerance to fluconazole [24,31]. Independently of the nature of mechanisms for different responses of WT and rho^−^ mutants, enhanced tolerance to antifungals could be judged as an advantage of the yeast petites for survival in the presence of at least some antifungals. Such an advantage seems to be particularly universal regarding petites of *Saccharomyces* species growing in the presence of fluconazole. Potentially, the tolerance could be considered as a genetic marker for respiratory deficiency including not only rho^−^ mutations, but also nuclear mutations affecting mitochondrial functions. For example, mutations in the nuclear *ERG3* gene of *S. cerevisiae* led to azole resistance [24], while the deletion of nuclear gene *PIF1* in *S. cerevisiae* caused petite phenotypes [32] and enhanced tolerance to clotrimazole and fluconazole (Supplementary Figure S1).

In this study, we clearly demonstrated the possibility of the positive selection of mutants with dysfunctional mitochondria in an experiment where all resistant to fluconazole colonies originated from the WT population of *S. cerevisiae* were respiratory-deficient as they did not grow on YEPG medium with glycerol as a sole carbon source. Such selection is not invasive and robust, and it allows for the monitoring and quantification of all events of respiratory deficiency at a large populational scale. All selected clones are alive; they could be stored and analysed further for various properties.

The positive selection could be useful for the discovery of nuclear mutations suppressing the petite-negative status of certain yeast species. Such suppression has been described for *Schizosaccharomyces pombe* [33]. The easiness of monitoring respiratory deficiency would allow for wide genome screen experiments using gene knockout collections to discover multiple nuclear genes affecting the petite-negative status as well as the frequency of spontaneous mitochondrial mutations in petite-positive species.

The effective positive selection of respiratory-deficient mutants could be utilised to solve different tasks. First, it provides an approach to accurately analyse the frequencies of mutations of mitochondrial deficiency in natural yeast populations. It could also be used as a method for selecting mitochondrial mutants without mutagens, in contrast to using ethidium bromide [9,33,34,35]. Presumably, using different concentrations of fluconazole (or other antifungals) would permit the “sieving” of various genetic yeast variants correlating with different levels of resistance to selective agents. Since no induced mutagenesis is involved, any discovered differences between isolates are likely to reflect natural biological processes within yeast populations that are driven by a high mutability of mitochondrial DNA [36].

As a genetic marker, tolerance/resistance to antifungals (e.g., fluconazole) could be used to manipulate the inheritance (or retention) of rho^−^ cytoplasm in genetic crosses and experiments involving genetic transformation. This will be a new tool for studying nucleo–mitochondrial interactions in yeast [37] and could be also used for selective retaining of rho^−^ cytoplasm in the production of somatic hybrids by protoplast fusions [38,39,40,41].

Mitochondrial mutants are expected to have reduced fitness and adaptivity to stress factors in comparison to their WT counterparts [12,15,16,18,19], thus implying the importance of mtDNA in at least some processes of adaptive evolution. Enhanced tolerance of rho^−^ mutants to antifungals implies their better performance compared with the WT in certain experimental or natural environments. In another example of this sort, cells of *S. cerevisiae* petites manifested resistance to cobalt sulphate three times that of the WT cells [42].

The observation of different ZOI patterns for the incomplete inhibition of the WT (Figure 6) is consistent with the presence of some proportion (sometimes substantial) of petite mutants generated spontaneously in the WT populations and accumulated during their cultivation on a fermentable substrate (such as YEPD medium in our case). Since mitochondrial mutants are more tolerant to antifungals than WT, their minimum inhibitory concentrations (MICs) are higher than the MICs for WT. Therefore, rho^−^ mutants form colonies within the areas of ZOIs formed by non-replicative WT cells. The ZOI pattern of the E-type takes place if a rho^−^ mutant is highly tolerant (practically resistant) to an antifungal and can grow equally well at high and low drug concentrations around the disc. The alternative C-type is characteristic for populations of cells with the same MIC, and thus with a clear boundary of the ZOI. The P-type pattern is generated when a rho^−^ mutant is tolerant to low drug concentrations at the periphery of the ZOI for WT but sensitive to higher concentrations in ZOI areas close to the disc. T-type ZOI patterns could be produced as a result of P-type scenarios combined with the actions of efflux pumps that are induced by some drugs in certain microorganisms. In conjunction with the peculiarities of drug diffusion from the disc and the extent of microbial growth, efflux pump processes reallocate local drug concentrations within the ZOI, which resulted in inhibition or promoted the growth of a microorganism at an unexpected distance from the disc. Such mechanisms have been discussed for *Enterococcus faecalis* inhibited by triclosan and forming T-type like ZOIs [43].

Observing different modes of inhibition for the WT (mainly fungicidal) and rho^−^ mutants (predominantly fungistatic) provided an extended characterisation of the tolerance of yeast species to antifungals. Using replica plating of inhibited cells to fermentable and non-fermentable media could be a potentially new simple approach for testing the MoI. Interestingly, replica plating was initially proposed as an indirect method for selecting bacterial auxotrophic mutants [26], but in our application, it could be interpreted as a method for the direct selection of rho^−^ mutants by utilising their property of resistance to antifungals.

## 5. Conclusions

Compared to the WT, mitochondrial rho^−^ mutants of five petite-positive species (including *C. glabrata*, *S. bayanus*, *S. cerevisiae*, *S. paradoxus*, and *S. pastorianus*) were more tolerant to at least two or more of the following antifungals: amphotericin B, clotrimazole, fluconazole, and miconazole. In particular, rho^−^ mutants of all *Saccharomyces* species were highly resistant to fluconazole, and *C. glabrata* rho^−^ mutants were the most tolerant to clotrimazole. We propose considering the enhanced tolerance to antifungals as a generic feature, allowing for selective advantages of respiratory-deficient cells over the WT at a population level. These findings imply different applications in genetics related to (a) the isolation of mitochondrial and nuclear mutants defective in respiration; (b) the use of resistance to antifungals as a genetic marker in sexual crosses and parasexual hybridisation of yeasts; and (c) estimating the rates of respiratory-deficient mutants in natural and experimental yeast populations.

## Figures and Tables

**Table 1:** Yeast strains used in the study.

Strain	Description/Genotype	Origin
***Candida glabrata***		
NCYC 3303	the strain deposited in 2005 by C. Bond (NCYC) {91} ^#^	NCYC *
Cg 3303-2	*rho* mutant induced with ethidium bromide {92}	**
Cg 3303-7	an individual wild-type clone (respiratory proficient) {97}	**
Cg 3303-8	an individual wild-type clone (respiratory proficient) {98}	**
***Saccharomyces bayanus***		
NCYC 106	the strain deposited in 1920 by A. Klocker (Carlsberg Laboratories, Denmark) as *Saccharomyces willianus*	NCYC
Sb106-1	spontaneous *rho* mutant of NCYC 106 {21}	**
Sb106-3	an individual wild-type clone of NCYC 106 (respiratory proficient) {29}	**
Sb106-4	an individual wild-type clone of NCYC 106 (respiratory proficient) {30}	**
Sb106-8	*rho* mutant induced with ethidium bromide {27}	**
***S. cerevisiae***		
DLY 640	*MATa ade2-1 trp1-1 can1-100 leu2-3,112 his3-11,15 ura3 GAL + psi+ ssd1-d2 RAD5* (wild type, W303 background, from R. Rothstein) {42}	D. Lydall
DLY 641	*MATalpha ade2-1 trp1-1 can1-100 leu2-3,112 his3-11,15 ura3 GAL+psi+ ssd1-d2 RAD5* (wild type, W303 background, from R. Rothstein) {40}	D. Lydall
DLY 2416	respiratory-deficient mutant generated by treatment of DLY 640 with ethidium bromide and proved to be a *rho* mutant {43}	**
DLY 2419	respiratory-deficient mutant generated by the treatment of DLY 641 with ethidium bromide and proved to be a *rho* mutant {41}	**
***S. paradoxus***		
NCYC 2910	the strain deposited in 1999 by Ed Louis (University of Oxford, UK)	
Spar 2910-1	*rho* mutant induced with ethidium bromide {115}	**
Spar 2910-2	*rho* mutant induced with ethidium bromide {116}	**
Spar 2910-3	*rho* mutant induced with ethidium bromide {117}	**
Spar 2910-4	*rho* mutant induced with ethidium bromide {118}	**
Spar 2910-7	an individual wild-type clone (respiratory proficient) {121}	**
Spar 2910-8	an individual wild-type clone (respiratory proficient) {122}	**
***S. pastorianus***		
NCYC 75	the strain deposited in 1920 by A. Klocker (Carlsberg Laboratories, Denmark) as *Saccharomyces carlsbergensis*	NCYC
Sp75-1	spontaneous *rho* mutant of NCYC 75 {1}	**
Sp75-2	spontaneous *rho* mutant of NCYC 75 {2}	**
Sp75-3	an individual wild-type clone of NCYC 75 (respiratory proficient) {7}	**
Sp75-4	an individual wild-type clone of NCYC 75 (respiratory proficient) {8}	**

* See website of the National Collection of Yeast Cultures: https://www.ncyc.co.uk/. ** These strains were characterised and described in a previous work [16]. ^#^ The numbers in braces correspond to records in our catalogue for frozen strains.

## Data Availability

The original contributions presented in this study are included in the article/Supplementary Material. Further inquiries can be directed to the corresponding author.

## Supplementary Materials

The following supporting information can be downloaded at: https://www.mdpi.com/article/10.3390/microorganisms13010099/s1.

## Abbreviations

DMSO	Dimethyl sulfoxide
*MATa*	Mating type a
*MATalpha*	Mating type alpha
MoI	Mode of inhibition
mtDNA	Mitochondrial DNA
NCYC	National Collection of Yeast Cultures
OXPHOS	Oxidative phosphorylation
*PIF1*	[from: Petite Integration Frequency] A nuclear gene encoding DNA helicase functioning in nucleus and mitochondria
*RAD5*	[from: RADiation sensitive] A nuclear gene encoding a DNA-dependent ATPase and DNA helicase
SEM	Standard error of the mean
W303	A genetic background of a yeast model, derived from K6001 background
WT	Wild type
YEPD	Yeast extract peptone dextrose medium (dextrose = glucose)
YEPG	Yeast extract peptone glycerol medium
ZOI	Zone of inhibition
ZOIs	Zones of inhibition
