# Supplementary material for: Enhanced Tolerance to Antifungals as a General Feature of Rho− Mutants in Yeast Species: Implications to Positive Selection of Respiratory Deficiency
Source: Microorganisms. 2025 Jan 7;13(1):99. doi: 10.3390/microorganisms13010099 (PMC11767389; doi:10.3390/microorganisms13010099)
Supplement: Supplementary file 1 [file microorganisms-13-00099-s001.zip › microorganisms-3373510-supplementary.pdf]

Article

Enhanced Tolerance to Antifungals as a General Feature of Rho⁻ Mutants in Yeast Species: Implications to Positive Selection of Respiratory Deficiency

Zachary Johnson, Farhan Nadim and Mikhajlo K. Zubko *

Centre for Bioscience, Manchester Metropolitan University, John Dalton Building, Chester Street, Manchester M1 5GD, UK; zacharyjohnsn@gmail.com (Z.J.); farhannadim72@outlook.com (F.N.)
* Correspondence: author: m.zubko@mmu.ac.uk

Supplementary Materials

Supplementary Table S1. Modes of inhibition of yeast species by four antifungals (concluded from replica plating assays depicted in Supplementary Figures 6 -10).

**Species**	**Modes of inhibition by four antifungals**			
	**Amphotericin B**	**Clotrimazole**	**Fluconazole**	**Miconazole**
C. glabrata	WT: cidal<br>rho⁻: NC*	WT: cidal<br>rho⁻: static	WT: static<br>rho⁻: static	WT: cidal<br>rho⁻: static
S. bayanus	WT: cidal<br>rho⁻: NC	WT: cidal<br>rho⁻: static	WT: static<br>rho⁻: NC	WT: cidal<br>rho⁻: NC
S. cerevisiae	WT: cidal<br>rho⁻: cidal	WT: cidal<br>rho⁻: static	WT: cidal<br>rho⁻: NC	WT: cidal<br>rho⁻: static
S. paradoxus	WT: NC<br>rho⁻: NC	WT: NC<br>rho⁻: NC	WT: static<br>rho⁻: NC	WT: cidal<br>rho⁻: cidal
S. pastorianus	WT: cidal<br>rho⁻: cidal	WT: cidal<br>rho⁻: static	WT: static<br>rho⁻: NC	WT: cidal<br>rho⁻: static

*NC – not concluded. In some cases it was not possible to conclude because of small ZOIs or no inhibition at all.

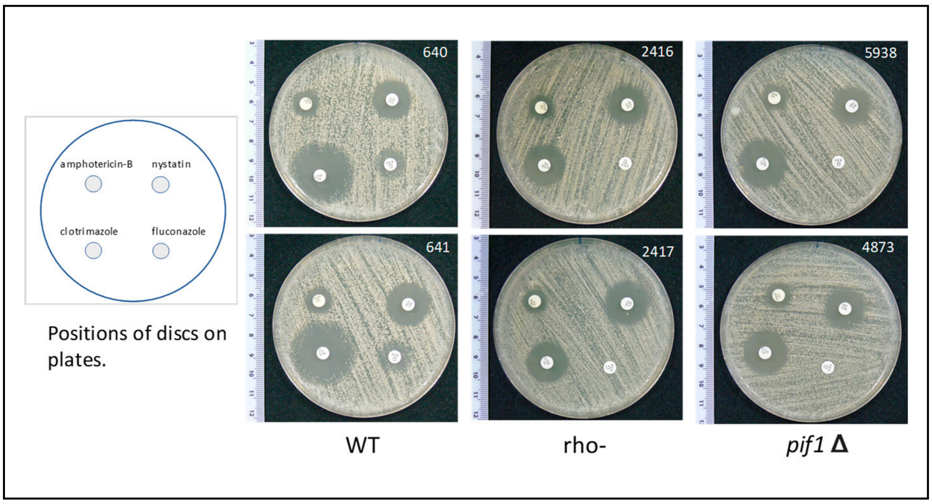

Supplementary Figure S1. Sensitivity responses of wild type and two classes of mutants of budding yeast to four antifungals in disc diffusion assays. Wild type (WT) and the mutants are represented by two isogenic strains of independent origin (their DLY numbers are indicated at the top right corners). The discs contained the antifungals in the following amounts: amphotericin B - 20 µg, nystatin – 100 µg, clotrimazole – 10 µg, fluconazole – 25 µg. Resuspended in sterile water cells were swabbed onto YEPD plates, and discs with antifungals were placed in the order shown in the scheme at the left. Plates were incubated at 25°C for 3 days. The pictures were taken with a ruler at the side to show sizes of ZOIs. (For more methodological details see Methods).

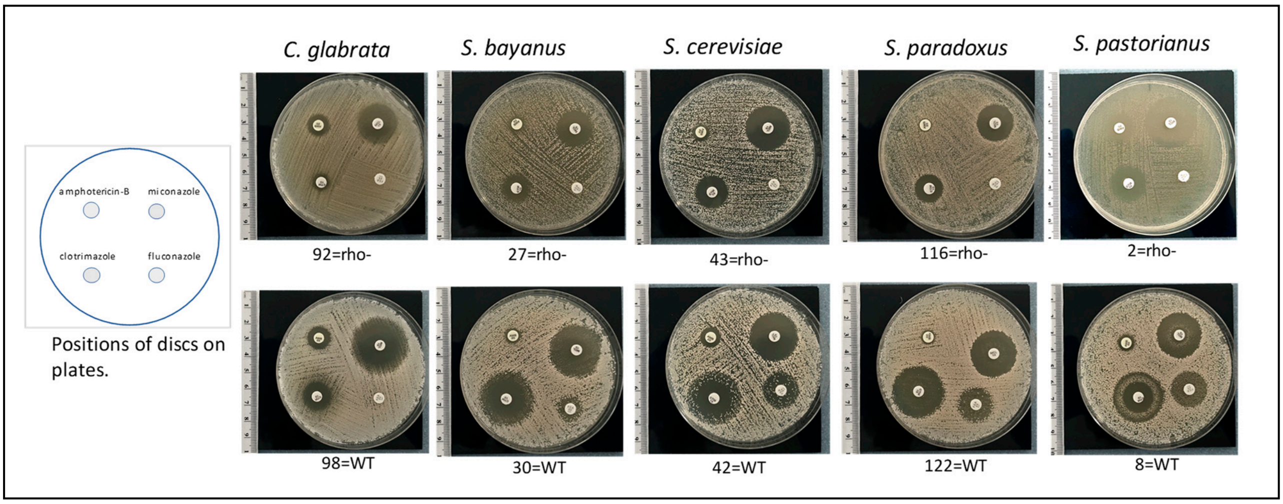

Supplementary Figure S2. Inhibitory effects and ZOI patterns produced by WT and rho- mutants of five yeast species in response to four antifungals in disc diffusion assays. Only one of two (or more) isogenic strains of independent origin is presented as a characteristic example for each species. The catalogue strain numbers under each image correspond to those in the description of the strains (see Table 1).

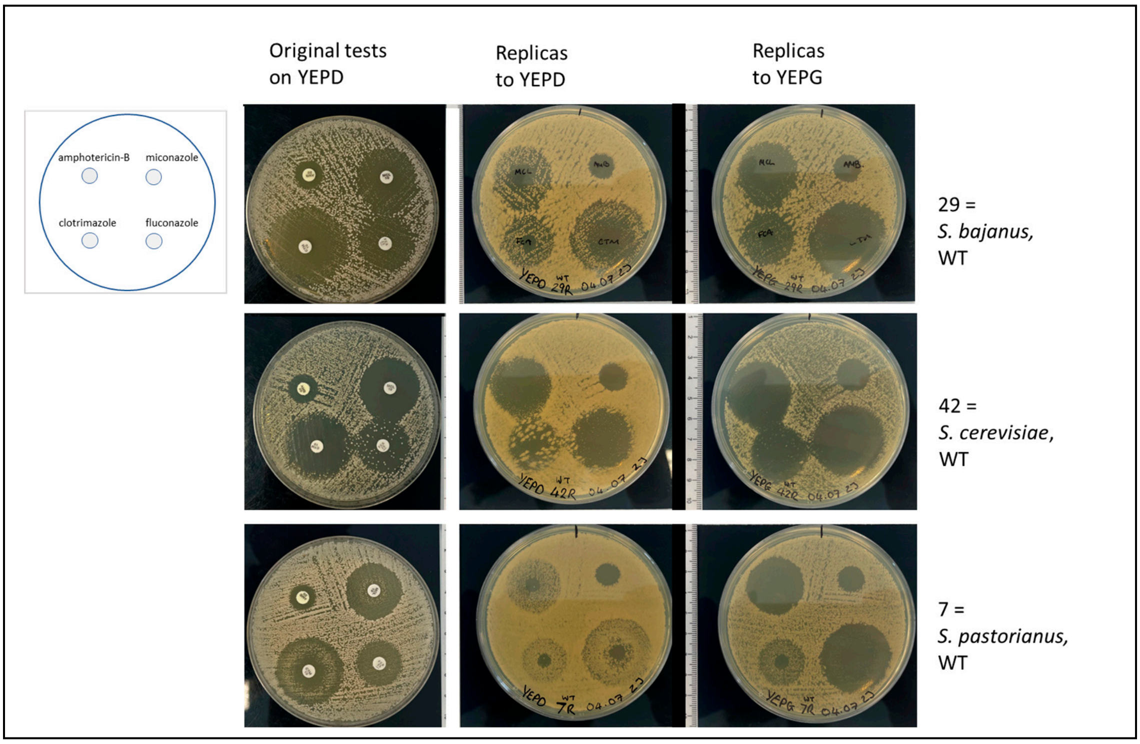

Supplementary Figure S3. Using replica plating for testing rho status of colonies emerging within ZOIs of WT yeast populations. Colonies which do not produce growth of their cells replicated to YEPG medium are derived from rho- cells.

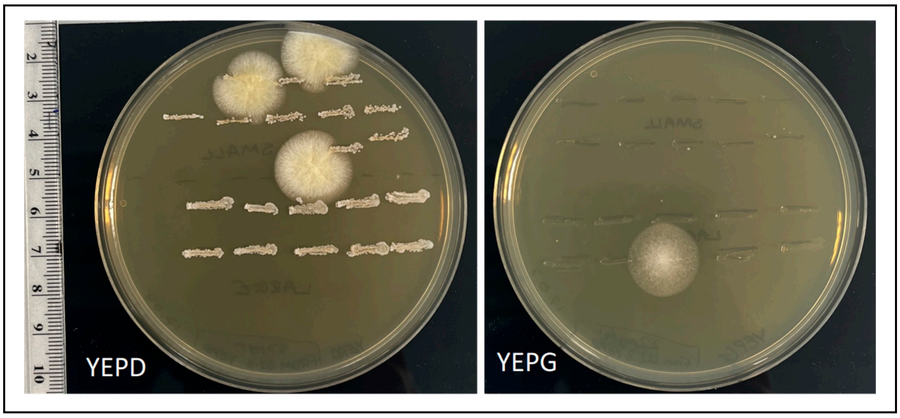

Supplementary Figure S4. An assay confirming respiratory deficiency of colonies emerged after plating WT *S. cerevisiae* cells on YEPD medium supplemented with 32 µg/ml fluconazole. 20 colonies of *S. cerevisiae* shown in Fig. 7 (10 small and 10 larger in size) were individually resuspended in 50 µl of water and patched in equal amounts onto YEPD and YEPG media. Despite fungal contamination was apparent after keeping plates for a few days, it did not affect the overall conclusion about respiratory deficiency of all colonies, based on the lack of growth on YEPG medium.

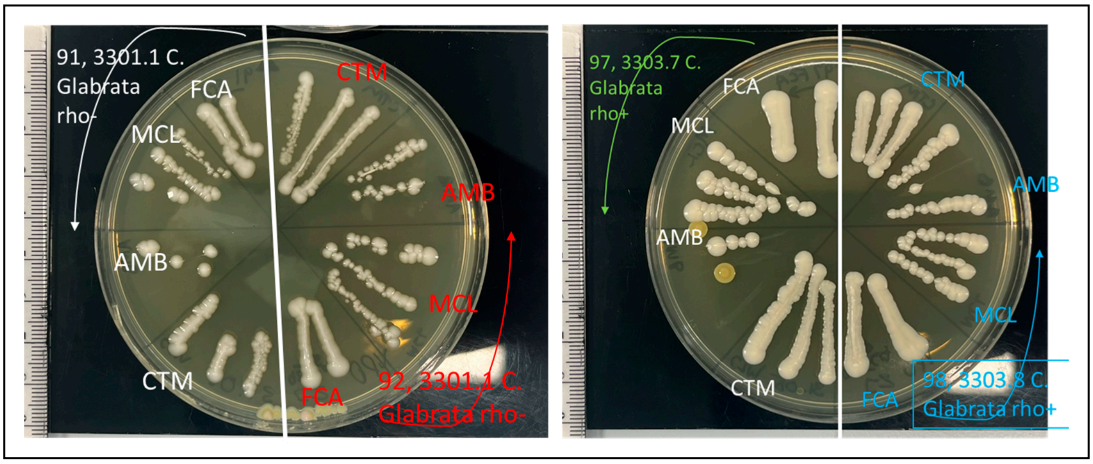

Supplementary Figure S5. Examples of streaking out from ZOIs for determining modes of inhibition for WT and a rho- mutant of *C. glabrata*. Streaks were taken from clean areas of ZOIs free of visually detectable colonies. Abundant growth from the streakes indicates fungistatic inhibition; poor growth or no growth are consistent with fungicidal effects. On the images, only amphotericin B shows signs of fungicidal MoI.

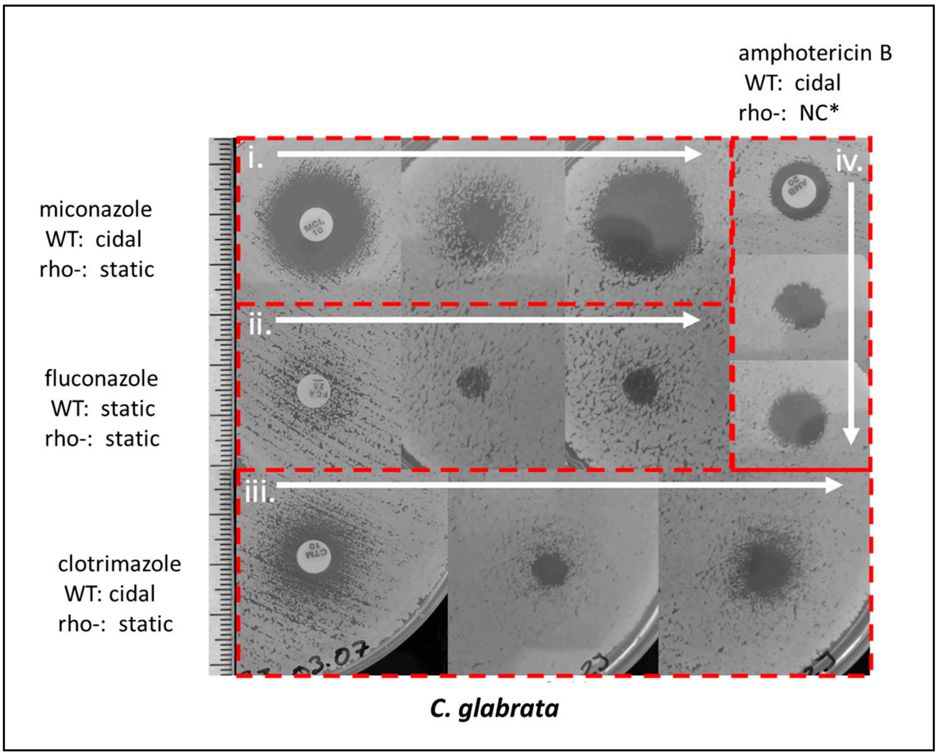

Supplementary Figure S6. Inhibition zones produced by WT *C. glabrata* (strain Cg 3303-8) for miconazole (i), fluconazole (ii), clotrimazole (iii), and amphotericin B (iv), with subsequent replica plating onto YEPD and YEPG. Directions of replica plating are indicated by arrows. Results are representative of both isogenic wild-type strains. NC* - not conclusive.

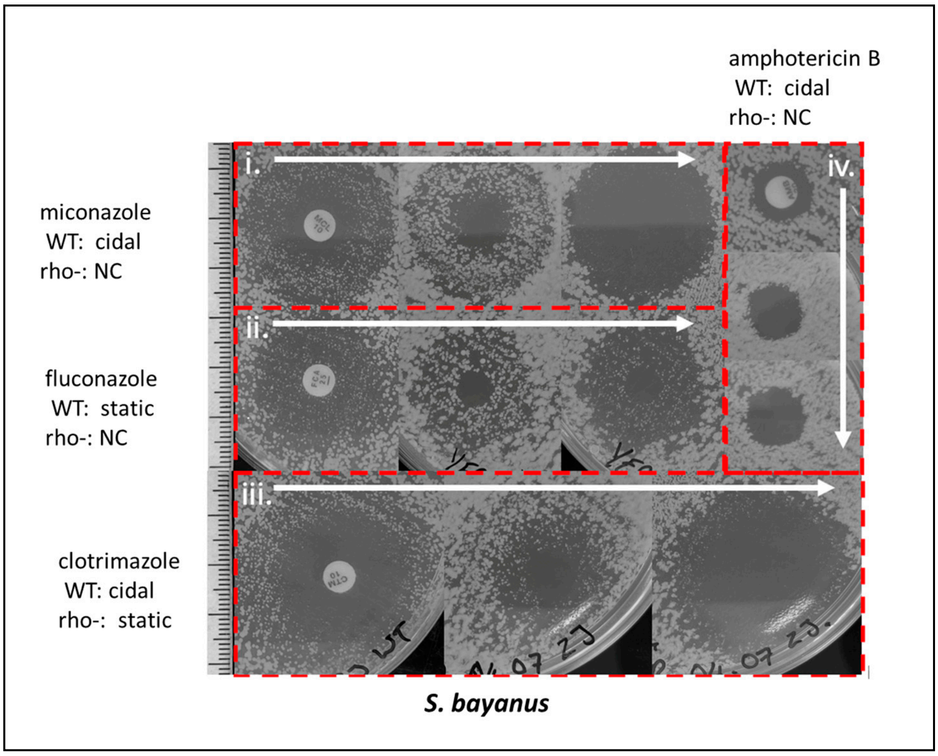

Supplementary Figure S7. Inhibition zones produced by WT *S. bayanus* (strain Sb 106-4) for miconazole (i), fluconazole (ii), clotrimazole (iii), and amphotericin B (iv), with subsequent replica plating onto YEPD and YEPG. Directions of replica plating are indicated by arrows. Results are representative of both isogenic wild-type strains.

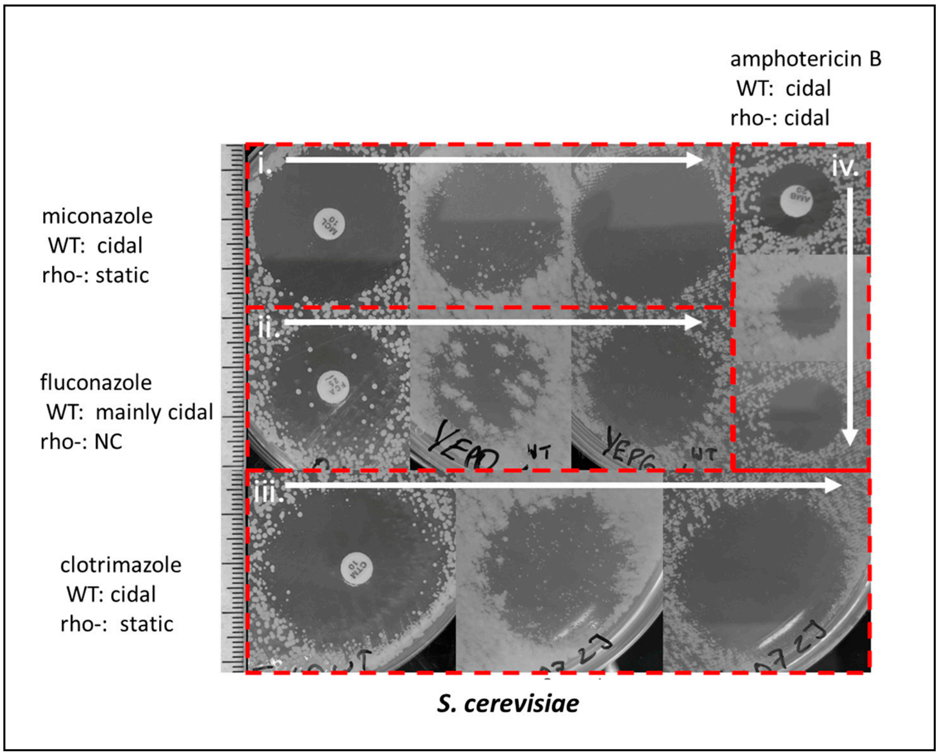

Supplementary Figure S8. Inhibition zones produced by WT *S. cerevisiae* (strain DLY 641) for miconazole (i), fluconazole (ii), clotrimazole (iii), and amphotericin B (iv), with subsequent replica plating onto YEPD and YEPG. Directions of replica plating are indicated by arrows. Results are representative of both isogenic wild-type strains.

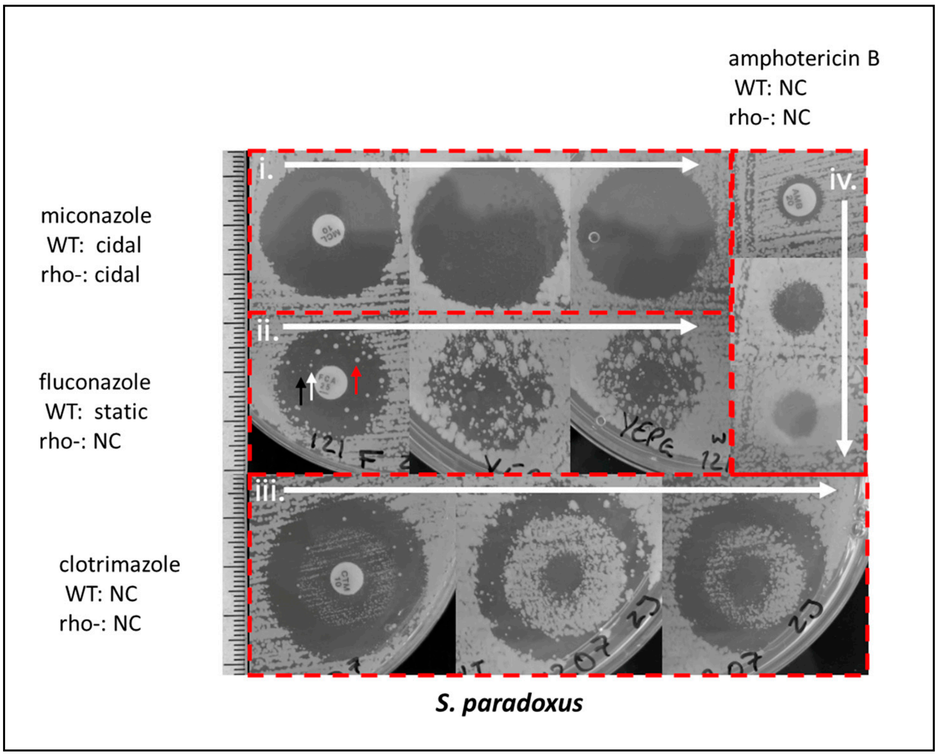

Supplementary Figure S9. Inhibition zones produced by WT *S. paradoxus* (strain Spar 2910-7) for miconazole (i), fluconazole (ii), clotrimazole (iii), and amphotericin B (iv), with subsequent replica plating onto YEPD and YEPG. Directions of replica plating are indicated by arrows. Results are representative of both isogenic wild-type strains. Black, white, and red arrows indicate small grey, medium beige, and large beige colony phenotypes, respectively.

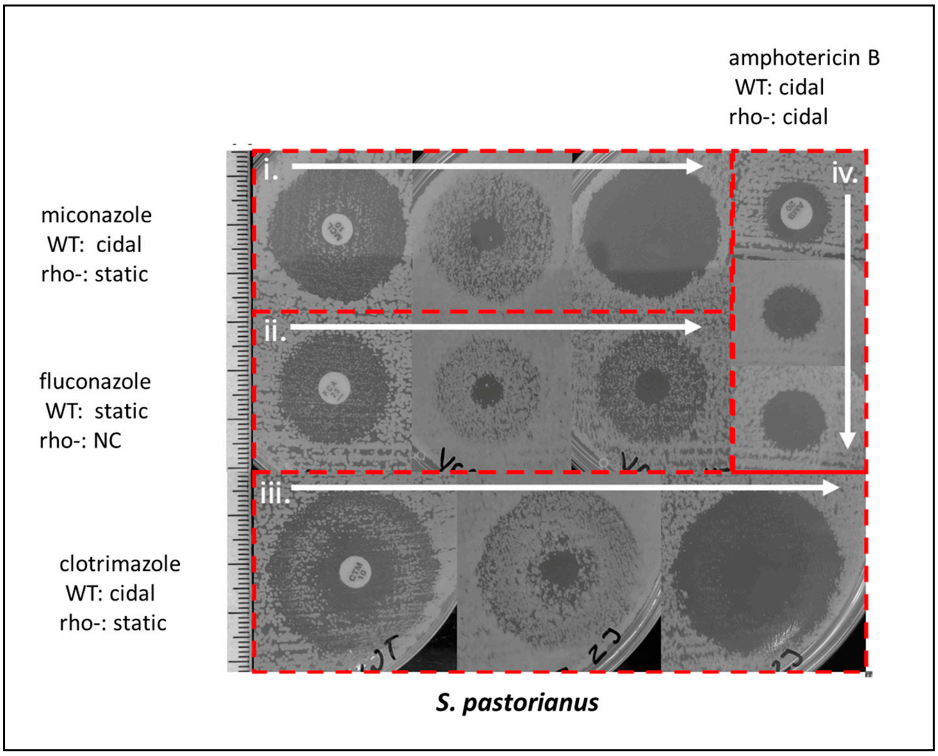

Supplementary Figure S10. Inhibition zones produced by WT *S. pastorianus* (strain Sp 75-3) for miconazole (i), fluconazole (ii), clotrimazole (iii), and amphotericin B (iv), with subsequent replica plating onto YEPD and YEPG. Directions of replica plating are indicated by arrows. Results are representative of both isogenic wild-type strains.

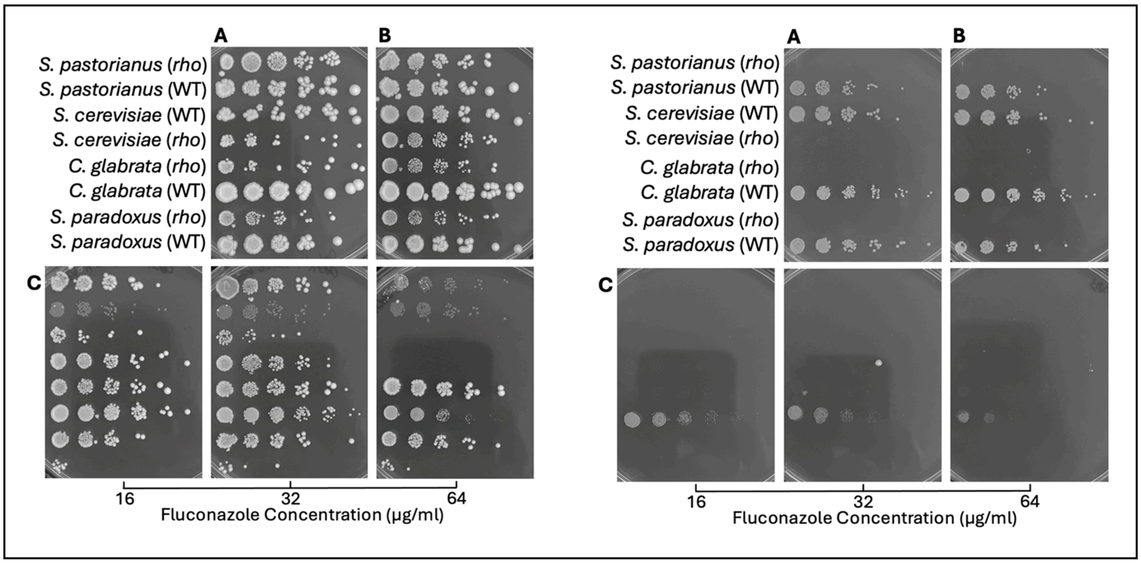

Supplementary Figure S11. Responses of WT and rho$^-$ mutants of four yeast species to three concentrations of fluconazole in YEPD (left) and YEPG (right) media. (**A, B**) – two replicas of the assay without fluconazole in the media; (**C**) – responses to different concentrations of fluconazole in the media.
